# Supplementary material for: Caregivers’ and Health Extension Workers’ Perceptions and Experiences of Outreach Management of Childhood Illnesses in Ethiopia: A Qualitative Study
Source: Int J Environ Res Public Health. 2021 Apr 6;18(7):3816. doi: 10.3390/ijerph18073816 (PMC8038672; doi:10.3390/ijerph18073816)
Supplement: Supplementary file 1 [file ijerph-18-03816-s001.pdf]

Supplementary materials (File S1) discussion and interview instruments

Guide for focus group discussion - Mothers in the community who have children less than 5 years of age will be the participants.

Explain the purpose of the study to obtain the consent from FGD participants

Region: Amhara

1. Zone: Awi
2. Woreda: **Dangila /Ankesha**
3. Kebele:___________
4. Village_____________
5. Name of facilitator__________
6. Name of recorder___________

Date of FGD_______________

Theme 1: Mothers 'awareness about availability of child health care services in their community

1. **Could you tell me about child health care services in your locality?**

PROBE:

Are you aware of the home based services provided by the HEW?

If yes what are the services they provide?

Theme 2: Mother's experiences with child care services at home

1. **Have you ever received any home/outreach service**

PROBE: If yes,

- What was the services provided to yours or someone else's child?
- Did the HEWs visit your home for outreach services?
- When the last time the HEW visited you, what was the reason for the visit? How did the HEW record it? Family health card? Registration book? Other?
- During home visit, have you ever received any medication immediately after diagnosis of illness

  If no? Why?
- What do you think is the advantage and disadvantage of receiving child health service at home? Why?

PROBE: If no,

What was the reason?

- (Never heard about service, too busy, provider was absent from the health facility)

Theme 3: Mothers' knowledge and care seeking behavior about child health problems

1. **Tell me about the time your child was sick, how did the illness start? What happened next?**

PROBE:

What was his/her symptoms?

Did you seek care outside home? Where?

Who made the decisions to take the child to the specified service site?

Why and how did you select that service site?

*If they didn't seek care **probe**,*

*Would you like to go to health facility (**health post**)?*

What was the reason for not seeking care?
(Severity of illness, location of the health facility, busy time of the caregivers Etc.

Theme 4: Mothers' preference for services

1. Where do mother usually take their sick child?
2. Where do you prefer to receive child health care services and why? HP? HC? Hospital? Home?

PROBE:

What do you think in the utilization of health by the people who live closer to the health post versus far way?

3. What care would you prefer the HEWs to provide to mother and children at home (as an outreach service) *(Take time to list down all the concerns and complaints)*

Theme 5: Summary

1. In your opinion what can be done better to further improve home base care to mother and children.

Log Sheet (FGDs)

Project Title				
Informant	Age (Year)	Educational Status	Religion	Number of children under five
P1				
P2				
P3				
P4				
P5				
P6				
P7				
P8				
P9				
Tape Label				
Context Note<br>about the circumstances of the FGD				
Substantive Note<br>(any issue that was not clear)				
Methodological Note<br>(any unexpected and unintended happening during the course of the FGD)				
Duration of the FGD				

Moderator: ________________

Note taker: ______________

Key informant interview checklist- HEWs

Explain the purpose of the study to obtain the consent from the key informants

Background information of key informant

SN	Age	Education level	Health post
1.			
2.			
3.			
4.			
5.			
6.			
7.			
8.			

Theme 1: Background

1. **What are your roles in this health post?**
2. **What do you do on day-to-day basis related to new born and child health care?**

Theme 2: HEW Experience on outreach service provision

1. **Do you provide the service as an outreach to mothers and children in their home?**

   If yes

   PROBE:

   What are these services?
   What kind of service you provide if the child was sick at home?
   What job aid do you take with you used to provide home based service?
2. **Could you describe your experience on service provision at community level since you start working as HEW?**
3. I am going to show you pictures (Back pack, foldable registration book, chart booklets, and treatment kits)) of intervention components back pack and foldable registration). When I show you the image I would like you to tell me whether you have seen it or not (**Interviewer note: keep away the images she has not seen and use only the images she has seen and probe on them**).
   A. Have you ever used it?
   B. How helpful do you think it was for your work?
   C. What do you think should be changed to improve the implementation of outreach services job aids?
4. **Describe the feasibility and sustainability of the service provision?**

   PROBE:

   Supply and system level

Theme 3: Barrier and enablers on service provision

1. **Please describe the barriers/ challenges you have faced in using backpack and other essential job aid tools during home visits?**

   PROBE:

   Can you tell me more about supply barriers and system barriers?
   General, barriers for outreach services:
2. **What are the enabler for your outreach services**

Theme 4: Community perception on home based services?

1. How do you describe community perception on this type of service you provide at home?

Theme 5: Community acceptability of home based service (Outreach)

1. **How do you describe the community acceptability of the provided treatment at home for their sick child?**
   PROBE:

   What changes have you observed at community level since you started providing service at their home?
   How do you describe service access using outreach, how is the service provision information disseminated?
   How do you describe community acceptability of the improved access due to outreach service?
2. **In your opinion, what are the difficulties that you face in providing outreach services?**
   PROBE:

   What do you think will help overcome them?

   Thank you, any question or comment.
